# Experimental and Simulation Investigation of an Adaptive Model Predictive Control Scheme: Model Parametrized by Orthonormal Basis Function

**DOI:** 10.1021/acsomega.3c09894

**Published:** 2024-01-19

**Authors:** Muddu Madakyaru

**Affiliations:** Department of Chemical Engineering, Manipal Institute of Technology, Manipal Academy of Higher Education, Manipal 576104, India

## Abstract

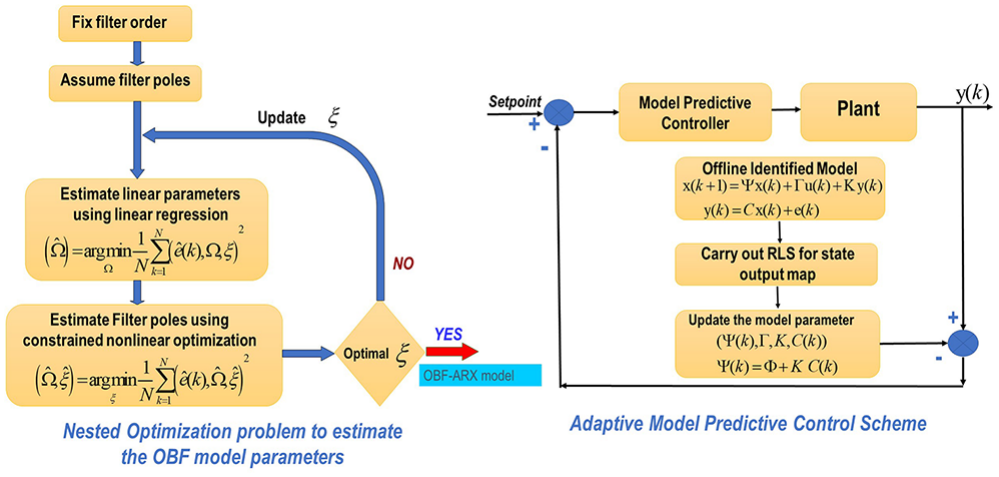

The closed-loop system’s performance in synthesizing model predictive control (MPC) heavily relies on the model used for prediction. In continuously operating plants, a linear model-based MPC is designed based on the operating point’s linear model during the commissioning stage. However, if the plant requires significant transitions from its normal operating point, the linear model-based MPC may not be effective. Therefore, to maintain the MPC performance under changing nominal operating conditions, the model (deterministic and stochastic components) needs to be updated to predict every sampling instant. This study focuses on designing an adaptive MPC (AMPC) scheme based on the linear model estimated from the input–output perturbation data under nominal operating conditions. The OBF–ARX (generalized orthonormal basis filters with ARX structure) parametrizes the observer’s dynamic components. The proposed fixed and variable pole AMPC schemes’ efficacy is demonstrated using a simulation study on a binary distillation column and experimental evaluation studies on a benchmark two-tank heater setup. The efficacy of the proposed AMPC schemes in addressing both servo and regulator problems has been demonstrated through simulation and experimental results. Specifically, these schemes have been shown to effectively track set points while simultaneously rejecting disturbances. These findings suggest that the AMPC schemes hold promise for use in a variety of applications in which precise control is required.

## 1 Introduction

The chemical process industry (CPI) involves intricate unit operations and processes that necessitate constant supervision and regulation. To address this challenge, model predictive control (MPC) was developed, which has significantly enhanced the operations of the CPI over the last 30 years.^1,2^ The efficacy of the online predictions of future plant behavior and closed-loop performance is determined by the models used in the MPC scheme. In any plant that operates continuously, MPC is established at the commissioning stage by integrating a linear model obtained under the nominal operating condition. This model is developed only once, near the operating conditions, at the commissioning stage. If a plant requires significant changes to be made, the linear model developed during the commissioning stage will become ineffective in predicting the plant’s behavior. The primary goal of MPC formulations is to address plant model mismatches and the presence of unmeasured disturbances. A large model plant mismatch can significantly degrade the closed loop controlled performance.

The stability and performance of linear MPC schemes face challenges with changing operating conditions.^2^ Robustness in the controller design can be difficult and conservative. Developing multiple linear models is costly due to production loss. Updating linear model parameters intermittently is the most practical solution. Industrial applications of MPC use linear empirical models from time series analysis.^1^ The model should capture dynamics from both known inputs and unmeasured disturbances for effective disturbance rejection. The true order of the unmeasured disturbance model is often unknown, but it can be chosen to be high. When modeling high-dimensional systems like packed-bed distillation columns or tubular reactors, high-order models are needed to avoid bias errors. The ARX and far-infrared (FIR) structures are the most commonly used in industrial applications. The ARX structure is attractive for modeling high-order or distributed parameter systems and capturing unmeasured disturbance dynamics of unknown order, but conventional ARX models require many parameters to be estimated.

It is worth noting that the variance errors in parameter estimates increase with the number of model parameters and decrease with the length of the data.^3^ As a result, the conventional ARX model requires a significantly large amount of data to maintain low-variance errors. However, if we can reparametrize the ARX model such that it requires fewer parameters during the identification stage, then we can reduce the length of data needed for model identification. This reduction in data length will result in reduced loss of production during the model identification process, and it will decrease the cost of intermittent model reidentification.

Chemical plants require a MPC scheme that is often paired with an online optimization scheme for supervisory purposes.^4−6^ The need to generate good predictions in the face of changing operating conditions and plant characteristics can be fulfilled by updating the linear model parameters online. However, this approach has not received much attention in the industrial applications of MPC. Ydstie^7^ identified the admissibility problem and the instability of the parameter estimator or parameter drift as key issues that must be addressed while developing an adaptive control scheme. An alternate approach is to use model parametrization, such as Laguerre or Kautz filter-based models,^7^ which guarantees that the identified model is well-behaved. If the ARX models are reparametrized using orthonormal basis filters (OBFs) like Laguerre/Kautz filters, then the parameter drift problems can be eliminated to some extent, and the admissibility problem can also be addressed.

The MPC controller’s design is based on process models initially developed. However, as the system moves away from the initial nominal operating condition, the deterministic model predictions become inaccurate. This creates a significant amount of plant model mismatch due to the varying process operating conditions. As a result, the model’s predictions no longer reflect the actual plant dynamics, leading to deteriorating controller performance and robustness. This can even destabilize the control loop. Moreover, the disturbance characteristics also change with the operating conditions. To maintain the optimal level of MPC performance in the presence of changing operating conditions, both the deterministic and stochastic components of the prediction model must be updated. Adaptive control systems provide flexible solutions for uncertain, nonlinear, and time-varying processes. They offer significant benefits for challenging control problems, where the process is poorly understood and changes unpredictably. These benefits have been demonstrated in many successful industrial applications.^8^

Several researchers have proposed methods for online or periodic updates of model parameters, recognizing the need for adaptive MPC (AMPC). One notable contribution is from Nikolaou and co-workers,^4,9^ who formulated and solved an optimization problem involving MPC formulation and identification approaches online. This approach addresses the issue of persistent excitation (PE) during minimal system disturbances necessary for online parameter estimation.^10^ However, the resulting parameter estimation problem is computationally exhaustive. To address this, Vuthandam and Nikolaou^11^ reformulated the PE problem in the frequency domain.

To account for plant-model mismatch in real time, Ohshima et al.^12^ proposed a method in which the ARX model is estimated using the residual signal. The parameters of the ARX model are then estimated online using a recursive least-squares (RLS) method. This approach facilitates updates of the deterministic and stochastic components online. However, using the FIR representation for the deterministic component and the ARX representation for the stochastic component requires a large number of parameters to be estimated online.

Mdoe et al.^13^ have devised a technique to attain recursive, feasible, and robust stability criteria by employing input to state practical stability in their study. They achieve a minimum stabilizing prediction horizon by employing sensitivity and terminal ingredients through parametric nonlinear programming (NLP). The efficacy of this approach is demonstrated through a numerical example. Griffith et al.^14^ address the issue of updating the predictive horizon online for adaptive nonlinear MPC (NMPC) through the evaluation of sensitivity using a NLP approach. The infinite horizon problem is approximated through a selection of terminal conditions. The resulting controller is found to be stable with respect to input-to-state practically stable, and it is observed that the stability constant depends on the magnitude of nonlinearity in the region. Boiroux et al.^15^ proposed an AMPC strategy for regulating glucose levels in patients with type 1 diabetes. The designed MPC consists of a deterministic and stochastic part. It is shown that a fixed second-order deterministic model and the adaptive nature of the stochastic part of the MPC can produce reasonable closed-loop results.

Karra et al.^6^ presented an AMPC strategy for multivariable time-varying systems in their study. Their approach involves designing two separate recursive pseudolinear regression methods to incorporate the deterministic and stochastic components of the model in an online setting. The output error (OE) structure is utilized to capture the deterministic component, while the residue obtained from the OE model is modeled to account for the unmeasured disturbances. A time-varying state-space model is formed and used in online prediction in MPC formulation by combining deterministic and stochastic models. The main advantage of this model lies in its ability to separate the stationary and nonstationary components of the unmeasured disturbances. Maiti and Saraf^16^ have proposed an online identification of impulse response coefficients, which are subsequently used in MPC formulation to control distillation columns.

The challenge involved in reducing the computation time required for MPC with many state constraints is addressed by Nouwens et al.^17^ In their study, the idea is to remove a subset of the state constraints at each time step to reduce computational complexity while ensuring the closed-loop behavior remains identical to the original MPC. Further, it introduces an approximate constraint-AMPC (ca-AMPC) scheme that further reduces computation time by balancing closed-loop performance and constraint satisfaction properties. Further, a MPC formulation for nonlinear continuous-time systems with bounded parametric uncertainty and additive disturbance is presented.^18^ This work reduces conservatism during online operation, guarantees robust constraint satisfaction, and converges to a neighborhood of the desired set point. Pereira et al.^19^ proposed a path-tracking controller for autonomous vehicles that ensures safe and comfortable operation while minimizing wear and tear. The controller’s stability is proven using Lyapunov techniques. They also suggest a novel model for the online adaptation of the controller response, estimated using Kalman filtering. The proposed approach is evaluated through simulations and experiments on a Scania construction truck, demonstrating its effectiveness.

An adaptive horizon multistage MPC algorithm is developed that reduces the computational cost in NMPC systems with uncertainty.^20^ The algorithm uses parametric NLP sensitivity and terminal ingredients to determine the minimum stabilizing prediction horizon. This approach decreases computational costs in complex optimization problems. An adaptive predictive control method based on the Laguerre function is proposed to improve the performance of autonomous underwater vehicles in complex hydrological conditions.^21^ The method consists of an AMPC module for accurate tracking and a Laguerre function module to reduce computations. The RLS algorithm is used for identifying the model parameters to enhance accuracy and robustness. The Laguerre function helps to reduce the matrix order of the objective function, limiting computation in complex environments.

Two noncooperative distributed AMPC (dAMPC) schemes are proposed based on ARX models parametrized using generalized OBFs (GOBFs).^22^ The efficacy of the proposed dAMPC schemes is demonstrated using simulation studies on an octuple tank process. Kumar et al.,^23^ developed an adaptive dual MPC scheme that uses generalized orthogonal basis filters to update model parameters online using a RLS algorithm. The approach uses state-space realizations of GOBF networks for model development and prediction, and simulation studies show promising results. Adetola and Guay^24^ developed a solution for robust AMPC for a class of uncertain nonlinear systems with state and input constraints using a novel set-based adaptive estimation.

The article presented the conditions for the recursive feasibility and stability of MPC applied to nonlinear systems with time-varying state constraints. These constraints could arise from changes in the environment, and the article provides conditions for guaranteed recursive feasibility and stability when the change in constraints is bound or when a prediction model is available. Additionally, the article details a robust multiscenario MPC technique suitable for processes with uncertain parameters or external disturbances.^25,26^ This approach assumes a finite number of possible values for the uncertainties and models their combinations in a scenario tree. The classical dual mode approach of nominal MPC is adapted to establish recursive feasibility and stability for the multiscenario case, using a standard terminal region and cost function for all uncertainty realizations.

The ARX and FIR structures are widely used model structures in the process industry for developing MPC strategies. One of the main advantages of these structures is that they are linear in parameters and have closed-form solutions for parameter estimation. This makes them highly suitable for applications where the accurate and efficient estimation of model parameters is essential. The ARX model structure, in particular, is capable of estimating both deterministic and stochastic components of a model simultaneously, making it a good option for modeling both stable and unstable systems. However, capturing both the deterministic and stochastic parts of the system requires a higher-order model with more parameters to estimate, which can lead to a nonparsimonious model.

To address this issue, the ARX model structure can be reparametrized using an OBF, which can significantly reduce the number of parameters to be estimated during the identification stage and online parameter updates. This, in turn, results in a significant reduction in the identification effort, making the model more efficient and easier to implement. Overall, an AMPC formulation that is carefully designed and implemented is expected to perform well in the presence of unmeasured disturbances and plant-model uncertainties. This can lead to better closed-loop control of industrial processes, improving plant efficiency.

The process dynamics are captured by parametrizing the model with GOBF’s. The resulting model is then used to make multistep predictions and formulate a control law within a linear MPC framework. The RLS method is used to update the state-output map parameters online at each sampling instant. Both fixed- and variable pole locations are used to account for changes in the plant’s operating conditions. An adaptive approach is necessary to achieve the desired performance, and the proposed AMPC performance schemes are demonstrated through simulation studies on a binary distillation (BD) column. To demonstrate the feasibility of using the proposed AMPC (fixed- and variable pole) schemes, experimental studies are conducted on a benchmark heater-mixer setup^27^

The main contributions are as follows:

- Develop a reparametrized ARX model using OBF–ARX for capturing dynamics with respect to deterministic as well as unknown inputs.
- Develop an adaptive control strategy using OBF–-ARX-based models for maintaining the closed-loop performance in the face of changing operating conditions and unmeasured disturbance.
- Demonstrate the effectiveness of the proposed model identification and AMPC strategies by conducting
  - Simulation studies on a high-purity BD column, which exhibits strongly nonlinear dynamics.
  - An experimental demonstration on the benchmark two-tank heater experimental setup

The article is structured as follows: Section 2 provides a comprehensive explanation of the methodology including the theoretical background of the proposed model parametrization and model parameter estimation schemes. Section 3 outlines the adaptive framework for the MPC. Section 4 presents the Results and Discussion on two case studies, i.e., simulation study on the BD column to evaluate the performance of the proposed AMPC and the experimental demonstration and evaluation of the proposed AMPC schemes on the two-tank heater setup. Lastly, the concluding remarks are presented in the final section, which summarizes the simulation analysis and practical demonstration.

## 2 Methodology

This section provides a detailed understanding of the model identification and formulation of the proposed AMPC. In order to gain a better understanding of this subject matter, we will delve into the mathematical background required for the identification process. After that, the proposed AMPC formulation, which is an essential aspect of the overall control strategy, is presented. It is crucial to have a clear comprehension of the mathematical background, as it plays a critical role in understanding the proposed formulation of AMPC. Therefore, the initial section will provide a comprehensive overview of the mathematical concepts that are necessary for the identification process, followed by a detailed presentation of the proposed AMPC formulation.

### 2.1 Preliminary Mathematical Background

Let us consider a SISO time series model to better understand the model development stage
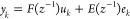
1

The given model consists of a zero mean white noise sequence denoted by {*e*_*k*_}. In order to estimate the parameters, the model is converted into a one-step predictor form
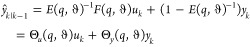
2

To evaluate the model parameter vector ϑ, an optimization problem is formulated with the objective function, as follows
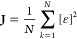
3where ε is defined as
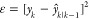
4

The process of estimating parameters using the prediction error method with a detailed description can be found in Ljung’s^3^ and Sodderstrom and Stoica’s^28^ works. This study proposes the development of *r* multiple input single output (MISO) models with an ARX structure. The approach of using GOBF’s is chosen to parametrize Θ_*u*_(*q*, ϑ) and Θ_*y*_(*q*, ϑ), which appear in eq 2. OBF are defined as per Ninness’s work^29^
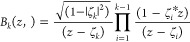
5

The set of filters {ζ_*k*_:*k* = 1, 2, ...} are represented by poles in complex conjugate pairs and have been shown by Heuberger et al.^30^ to be an OBF for the set of strictly proper stable transfer functions, denoted as . These filters are model parsimonious and described by a small set of parameters in the expansion, resulting in a strictly proper stable transfer. If the set of OBF has complex poles, Heuberger et al.^30^ have provided a method for state realization. In cases where time delays are known, additional poles can be introduced at the origin, apart from the GOBF poles.^31^

The right-hand side of eq 2 can be parametrized with OBF, as explained by Heuberger^30^
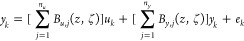
6

The MISO OBF ARX model, which pertains to the *i*’th output, can be represented in state realization form, as illustrated by Srinivasarao^32^
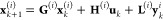
7
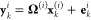
8

The mathematical model in question involves variables such as **x**_*k*_ ∈ *R*^*n*^ representing the states, **u**_*k*_ ∈ *R*^*m*^ representing the manipulated inputs, and {**e**_*k*_^*i*^} representing white noise. The predictor form, given by eqs 7 and 8, is a convenient way of representing the model and is often used during identification steps. The above model can be rearranged in the standard innovation form of the state space model as follows
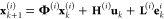
9
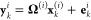
10where **Φ**^(*i*)^ = **G**^(*i*)^**+ L**^(*i*)^**Ω**^(*i*)^. The MISO model parameters obtained from identification exercises are referred to either as () or as ().

### 2.2 Model Identification: Model Parameter Estimation

**Figure 1:**
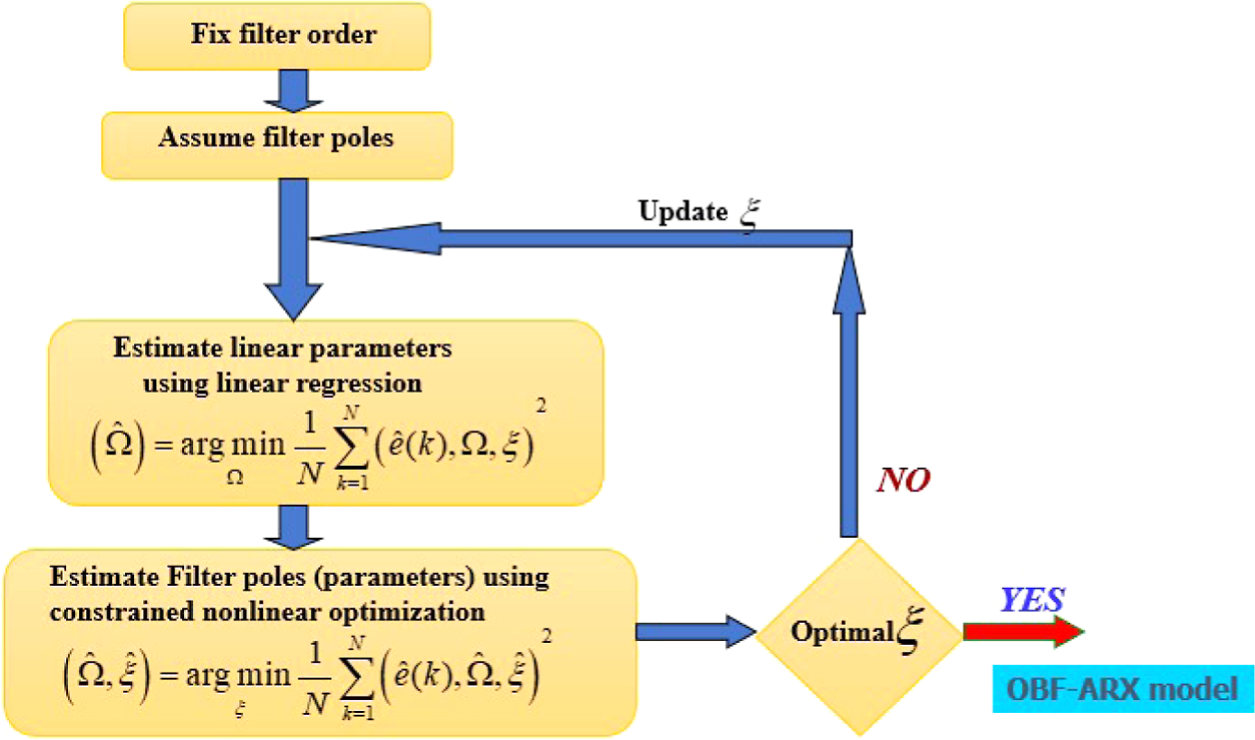
Nested optimization problem to estimate the parameters of the OBF model parameters.

The development of AMPC using the linear model exercise involves two steps. First, the model is identified from the perturbation data that is gathered offline by perturbing the plant around the nominal operating condition. Second, the state output map is updated online. To accurately represent the system dynamics, the challenge lies in determining the filter poles and the number of basis filters (filter order). To accomplish this, the first step is to assume the filter order and then search for the set of filter poles, as discussed by Srinivasarao et al.^32^ The model parameters are then estimated through two nested optimization problems, which are formulated based on the model order represented by the following methods and presented in the block diagram in Figure 1
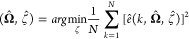
11subject to
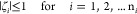
12Given, a guess of poles ζ, the parameter  is evaluated by solving another optimization problem
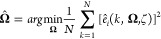
13The problem of estimating parameters can be resolved through the use of normal equations given the optimal poles for the GOBF (ξ)
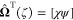
14where χ and ψ are as follows
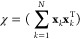
15
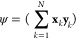
16

### 2.3 Online Model Parameter Estimation

This section delves into two distinct types of AMPC formulations: fixed pole AMPC and variable pole AMPC. To begin with, the offline model is first identified using perturbation data, which is valid in the vicinity of the operating point. However, in real-world scenarios, the plant’s behavior tends to fluctuate or drift in the vicinity of the operating conditions. In such cases, if these fluctuations or drifts are substantial, the prediction of the plant’s behavior from the model becomes inaccurate. Therefore, it becomes necessary to update the model online as operating conditions change to obtain accurate predictions under changing conditions. This is important to ensure that the system remains stable and safe while delivering an optimal performance. Hence, the online model adaptation technique is used to account for these changes and to update the predictive controller accordingly. This process ensures that the controller remains adaptive to the plant’s changing behavior, providing precise control actions even under uncertain and variable operating conditions.

It should be noted that the model’s state output map exhibits linearity in its parameters. To accommodate changes in operating conditions, the proposal is to employ an online RLS parameter estimation problem. The measurement equation is also revised to account for these changes in operating conditions, as shown in eq 17
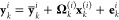
17

To account for time-varying and unmeasured disturbances on the output, the additive drift term  is introduced. The RLS estimation method is used to update the state output map of the model online. This involves updating  and **C**^(*i*)^. The *i*’th MISO model’s parameter and regressor vectors are determined using this approach^33^
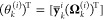
18
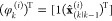
19where  represents the estimate of **x**_*k*_^(*i*)^, using the RLS approach, the parameter is updated as follows
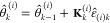
20
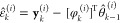
21
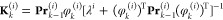
22
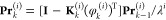
23

The Kalman gain matrix is denoted by **K**^(*i*)^, while the covariance matrix is represented by **Pr**^**(i)**^. The initial parameter vector is defined in the following manner
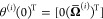
24

The initial value of **Pr**^**(i)**^**(0)** is determined through offline parameter estimation. The forgetting factor λ has a range of 0.95 ≤ λ ≤ 1. Two model parameter adaptation schemes can be obtained based on the form used to update **x**_*k*_^(*i*)^ in the regressor vector.

#### 2.3.1 Model Adaptation with Variable Poles

If the online state estimation can be carried out using the predictor form of MISO observer, i.e.
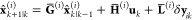
25
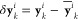
26then eq 25 transformed to innovation form
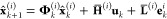
27where . In the MPC formulation, the innovation form is utilized to perform future trajectory predictions. It is worth noting that this transformation updates the system poles, which are defined by the eigenvalues of Φ_*k*_^(*i*)^, at each sampling instant.

#### 2.3.2 Model Adaptation with Fixed Poles

Another possibility is to update **x**_*k*_^(*i*)^ using the innovation form of state update equation of the form
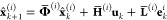
28where a fixed matrix  is used. Here,  is computed only once at the end of the initial off-line model identification exercise. By this approach, the poles of the model remain fixed.

### 2.4 AMPC Design

In this section, the formulation of AMPC for the variable pole case is presented. The formulation for the fixed pole case can be obtained by setting . Thus, at *k*th sampling instant, *r* innovation models of the form
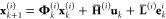
29
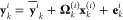
30where *i* = 1, 2, ..., *r* models are used to carry out the predictions. To get the combined state vector for a *r* × *m* MIMO process
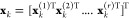
31

To obtain MIMO models, a viable approach is to combine *r* MISO models. This can be achieved by following a specific methodology
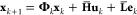
32
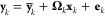
33where () are obtained by stacking .

### 2.5 Future Trajectory Predictions

To generate a model prediction for a future time window of [*k* + 1, *k* + *p*], by providing a guess for the future manipulated inputs. This can be achieved by using the set of inputs {**u**_(*k*+*i*|*k*)_:*i* = 0,1,2, ..., *p –* 1}
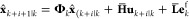
34
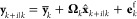
35
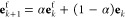
36
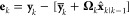
37

To reduce the impact of high-frequency noise on model predictions, use the tuning parameter α (where 0 ≤ α < 1). Furthermore, by incorporating the estimated initial state for the predicted horizon at the beginning of the process
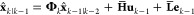
38

The input blocking constraints are imposed to shape the future trajectory
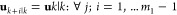
39
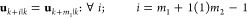
40
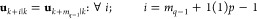
41

The *m*_*j*_ selected by putting the constraint as
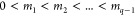
42where (*q*) and (*p*) are called as control horizon and prediction horizon, respectively.

### 2.6 Optimization Problem Formulation of AMPC with Time-Varying Terminal Weighting

In MPC, at a specific time instant, *k*, the problem is framed as a constrained optimization problem. The objective is to minimize an objective function while considering a set of future manipulated input moves, denoted by **U**_*k*_^f^ = {**u**_*k*|*k*_,**u**_*k*+1|*k*_, ..., **u**_*k*+*mq*|*k*_}. The manipulated input moves are computed by the optimization algorithm
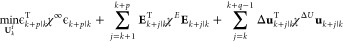
43subject to the following constraints
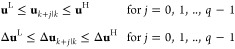
44where
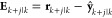
45
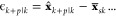
46
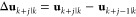
47

The two weighting matrices, namely, the symmetric positive semidefinite error weighting matrix denoted as χ^E^ and the symmetric positive definite input weighting matrix denoted as χ^Δ*U*^, are important components in the evaluation of the time-varying terminal weighting matrix, χk^∞^. This evaluation is done at each sampling instant using the discrete Lyapunov equation.^34^ The resulting output matrix is used to determine the optimal control parameters for the system under consideration
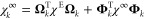
48

The terminal target state , which is time-dependent, is estimated as follows^34^
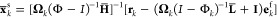
49

The MPC formulation mentioned above can be solved as a quadratic programming problem. However, only the first input move, represented by **u**_opt_(*k*|*k*), is executed on the plant during optimization, even though several input moves are produced. Additionally, the optimization problem is restructured at the next sampling time based on the latest measurements obtained from the plant.

## 3 Results and Discussion

Two case studies were conducted to assess the effectiveness of the proposed AMPC schemes. Case-study-01 presents the findings from a simulation study conducted on the BD column, while case-study-02 discusses the results obtained from the implementation of the proposed AMPC scheme on an experimental two-tank heater setup.

### 3.1 Case-Study-01: Simulation on Binary Distillation Column

**Figure 2:**
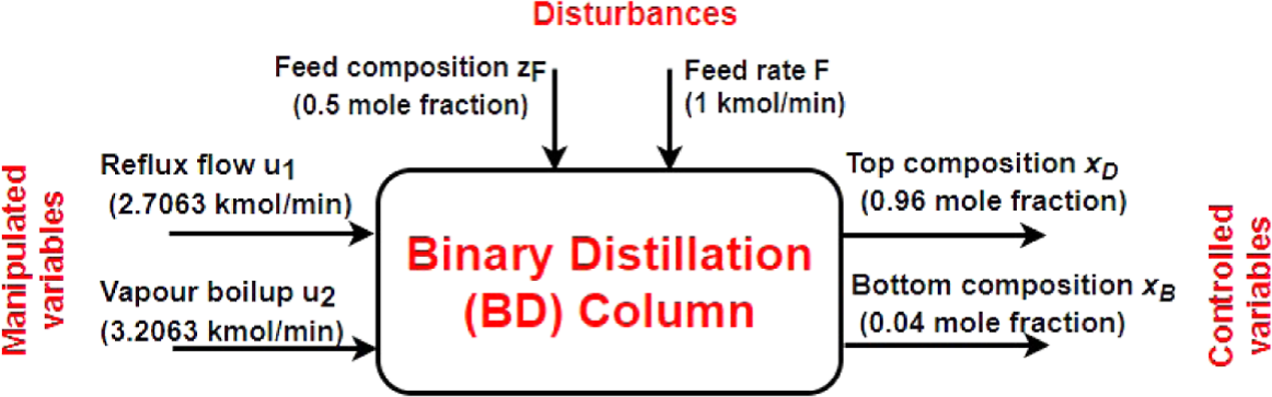
BD column: block diagram representing different variables and their nominal values.

The proposed AMPC scheme’s effectiveness was demonstrated through simulation on a BD column. The two-product BD column presented by Skogestad and Postlethwaite^35^ operated in a high-purity region. The details of the system dynamics can be found in Skogestad and Postlethwaite.^35^ The reflux flow (*u*_1_) and boil-up rate (*u*_2_) are used as manipulated variables to control the distillate product composition (*x*_D_) and bottom product composition (*x*_B_). In the present work, two variables are considered unmeasured disturbances, i.e., feed flow (*d*_1_) and feed composition (*d*_2_), which are random fluctuations around nominal values. The detailed block diagram representing the various variables and their nominal operating values is shown in Figure 2. The relative volatility value used (i.e., α = 1.4) is chosen so that it leads to higher separation with the same number of trays. Muddu^36^ has comprehensively explained the BD column’s operational conditions.

The dynamic simulation of the BD column is carried out using the program available from Skogestad and Postlethwaite.^35^ This process is described by 82 nonlinear coupled differential equations. The sampling time (*T* = 1 min) is used to solve the dynamic simulation.

### 3.2 Off-Line Model Identification

The reflux flow and boil-up rate are perturbed simultaneously using pseudorandom binary signals (PRBS) to obtain identification and validation datasets. The PRBS are utilized within the range [0, 0.022 ω_*N*_], where ω_*N*_ = π/*T* signifies the Nyquist frequency. The input signals are generated using the *idinput* function of the system identification toolbox of MATLAB. The measured variables, such as top and bottom compositions, are corrupted with zero mean Gaussian white noise sequences having a standard deviation of 0.005. The feed flow rate and composition are considered unmeasured disturbances and follow stochastic process dynamics
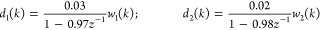
50

The given equation includes two white noise variables {*w*_1_(*k*)} and {*w*_2_(*k*)}, each with a standard deviation of 0.2 and 0.08, respectively. The perturbation exercises yield raw data with an absolute value, which is then scaled using their respective standard deviations (σ_*u*1_ = 0.0538, σ_*u*2_ = 0.0538, σ_*x*D_ = 0.0411, and σ_*x*B_ = 0.04440) to obtain zero mean unit variance. The dataset with zero mean unit variance is then used for model identification exercises.

A dataset of 2500 data points was collected, out of which the first 1500 data points were used to identify the model (identification dataset), and the remaining 1000 data points were used to validate the model (validation dataset). Two MISO models were developed from the perturbation data. During the development of the OBF-ARX models, two filter poles were utilized between each input–output pair **y**_*i*_ – **u**_*j*_. This resulted in 12 parameters that needed to be estimated for each MISO model. The optimal pole locations identified during the exercises are listed in Table 1. Instead of discrete pole locations (ξ_*i*_), the continuous time pole location (*a*_*i*_) is reported
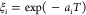
51where *T* represents the sampling time.

**Table 1:** BD Column: OBF–ARX Model Parameters (Optimum Pole Locations)

output	*u*_1_	*u*_2_	*x*_D_	*x*_B_
***x***_D_	[9.9965 9.9965]	[9.8785 9.9907]	[0.2228 0.2228]	- - - - - - - - - - -
***x***_B_	[9.9986 9.9986]	[9.9994 0.994]	- – – – – – – – -	[0.6733 9.5115]

To evaluate the performance of the identified model with validation data, the percentage prediction error (PPE)^37^ is utilized.

**Figure 3:**
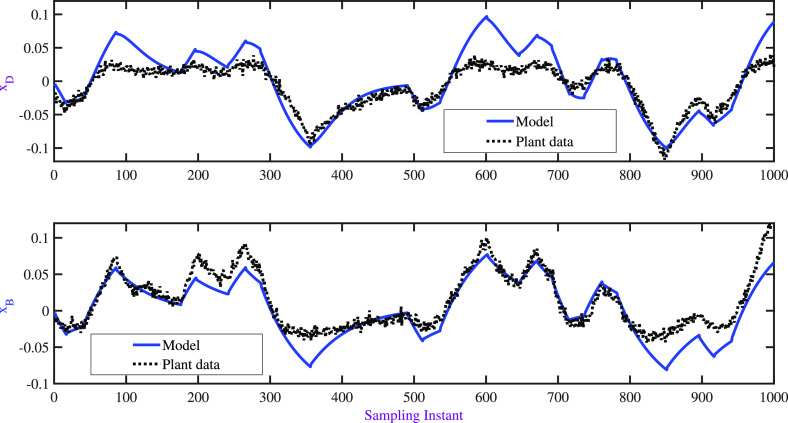
BD column: dynamic validation of identified model.

**Figure 4:**
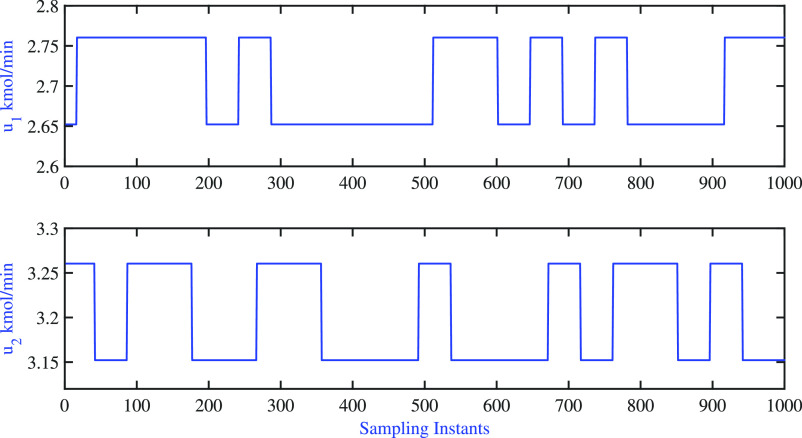
BD column: input moves are used for dynamic validation.

**Figure 5:**
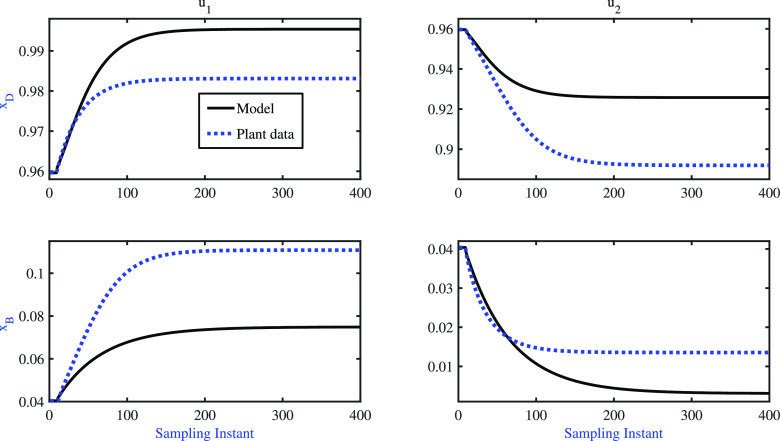
BD column: step response of the identified model.

**Figure 6:**
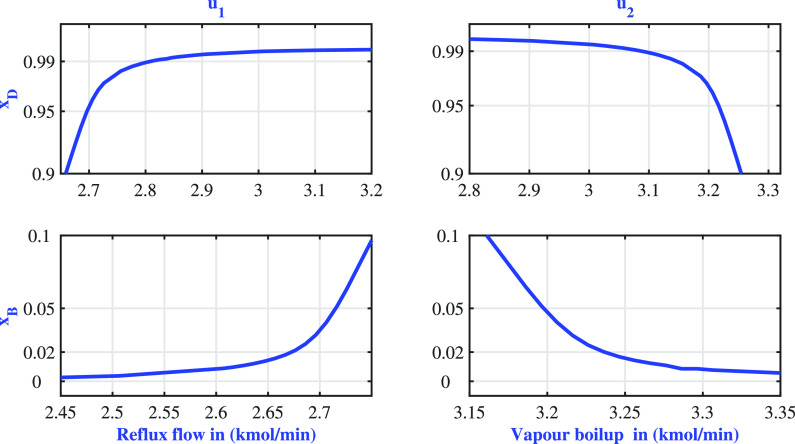
BD column: steady state input–output variations.

The effectiveness of the identified model was assessed by performing dynamic validation. The model prediction is shown in Figure 3, and the corresponding input moves are illustrated in Figure 4. The values of PPE for dynamic models **x**_**D**_ = 53.18 and **x**_**B**_ = 26.18 were observed to be significantly high. A better understanding of the identified model can be obtained by analyzing the step response of the model, which is depicted in Figure 5. The step response results reveal that the gain direction of the identified model matches that of the plant. However, a significant steady-state gain mismatch existed between the model and the plant. Furthermore, the simulation results of the dynamic model reveal significant discrepancies between the model predictions and plant outputs. This could be because the process is operating in a high-purity region. The variations in the steady-state behavior of the system outputs to the inputs are presented in Figure 6. Figure 6 indicates that the process operates under highly nonlinear operating conditions. The input excitation induces output variation in the following ranges
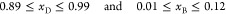


When operating a column close to or within the high-purity region, it is important to note that this is the area where nonlinear behavior becomes more prominent. This means that a linear perturbation model with fixed parameters may not be sufficient. To address this issue, the identified linear model fixes the time scale parameter (model poles) and can be used to initialize the online parameter adaptation.

### 3.3 Closed-Loop Simulations

This section will examine the effectiveness of the AMPC developed for BD simulation, and the performance is assessed using the following indicators.

Integral square error (ISE)
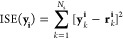
52

Performance index of the MPC objective function
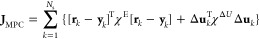
53where *N*_s_ is the number of sampling instants used for simulation.

**Figure 7:**
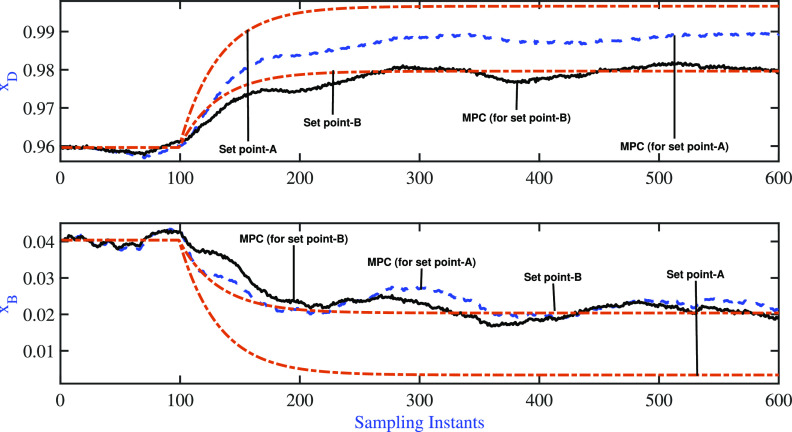
BD column: MPC: servo problem for two different set point—variation of controlled variable.

**Figure 8:**
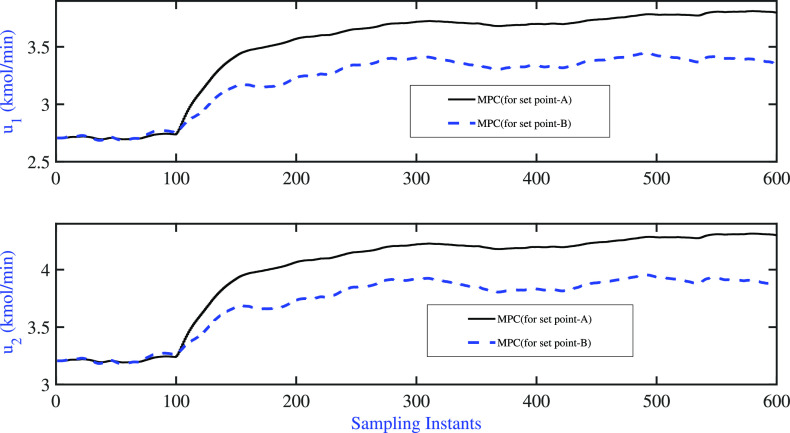
BD column: MPC: servo problem for two different set point—variation of manipulated variable.

Initially, a nonadaptive MPC scheme using a fixed parameter of the linear perturbation model was utilized to perform closed-loop exercises. The MPC tuning parameters for the BD simulation can be found in Table 2. The simulation study involved two set point changes of varying magnitudes. The closed-loop responses resulting from these changes are shown in Figure 7, and the corresponding manipulated input is presented in Figure 8. It is evident from Figure 7 that the fixed parameter-based MPC fails to generate acceptable servo behavior for large set point changes. However, for smaller changes in the set point, it is able to achieve a satisfactory response. Thus, the MPC with the fixed linear model works well in the small neighborhood of the nominal operating condition. However, to achieve better performance in the high-purity region, it becomes necessary to adapt the model parameters.

**Table 2:** BD Column: MPC Tuning Parameters

prediction horizon	50
control horizon	5
input blocking	[51051020]
input weighting matrix	**I**_2×2_
error weighting matrix	5 × **I**_2×2_
terminal state weighting matrix	0.001 × I2×2
filter coefficient	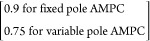

The proposed AMPC controllers (fixed- and variable pole AMPC) were evaluated through servo and regulatory experiments to assess their performance when the column is operated in the high-purity region. The AMPC tuning parameter used for the closed-loop study is presented in Table 2. The open-loop settling time of the BD column was found to be approximately 50 samples with a sampling time of 1 min, which formed the basis for the choice of the prediction horizon in MPC formulations. Since it is desired to achieve tight control of distillate composition as well as the residue composition, the error weighting matrix is chosen as 5 × **I**_2×2_, the control horizon is chosen as five, and these degrees of freedom are distributed over the prediction horizon using five input blocks of [5 10 5 10] samples each and the final input block of 20 samples.

**Figure 9:**
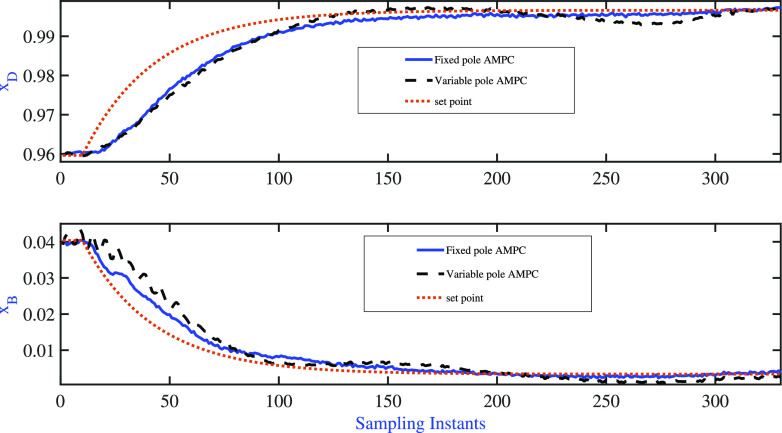
BD column: fixed pole AMPC and variable pole AMPC: servo problem—variation of controlled variable.

The servo simulation exercises were performed by introducing simultaneous step changes in **x**_D_ (from 0.96 to 0.9965) and **x**_B_ (from 0.0404 to 0.0034) at the 10th sample instant with the aim of moving the operation to the very high-purity region. The resulting variations of the controlled variable and the manipulated variable are listed in Figure 9. Both AMPC controllers were able to achieve the desired set point changes satisfactorily, and the performance indices (*J*_MPC_) and ISE values of the output for the servo problem are presented in Table 3. A comparison of the performance indices revealed that the fixed pole AMPC performed better than the variable pole AMPC during the desired set point change. The variation of model parameters during the servo problem was assessed in terms of the model sensitivity parameter, which is defined as
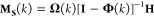
54

**Table 3:** BD Column: Comparison of Closed-Loop Performance Servo Problem

control scheme	*J*_**MPC**_	ISE(**x**_D_)	ISE(**x**_B_)
fixed pole AMPC	0.3897	0.0059	0.0017
variable pole AMPC	1.0397	0.0071	0.0052

**Figure 10:**
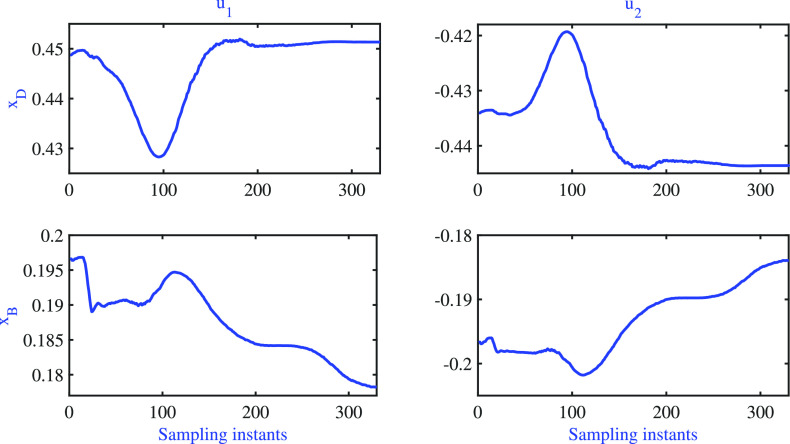
BD column: fixed pole AMPC: servo problem–variations of model sensitivity.

**Figure 11:**
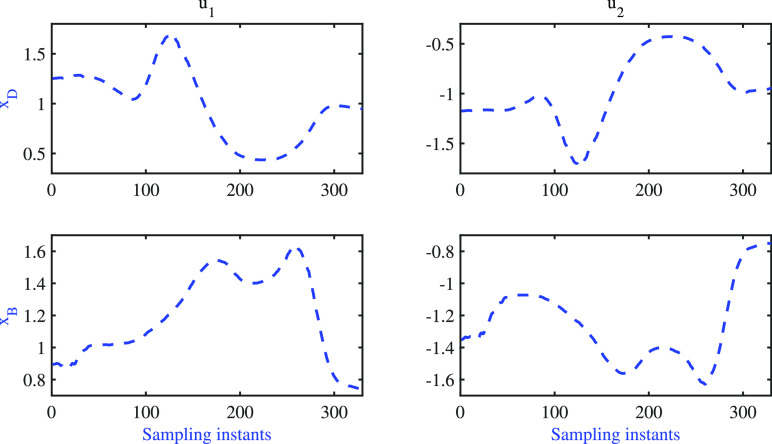
BD column: variable pole AMPC: servo problem—variations of model sensitivity.

The sensitivity matrix element variations for both fixed- and variable pole cases are presented in Figures 10 and 11, respectively.

**Figure 12:**
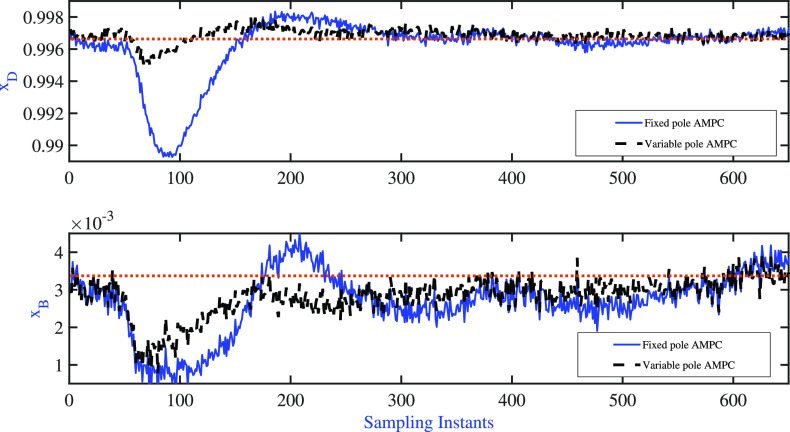
BD column: fixed pole AMPC and variable pole AMPC: regulatory problem—variations of controlled variable.

After the operation is shifted to the high-purity region, the adjustment of model parameters is halted. The closed-loop’s ability to reject disturbances is then tested at the new steady state by introducing simultaneous step changes in the feed flow (**d**_**1**_ = −20%) and composition (**d**_**2**_ = −20%) at the 50th sampling instant. The closed-loop responses of the controlled variables are shown in Figure 12. The performance of both AMPC strategies is tabulated in Table 4. The results indicate that the variable pole AMPC scheme can reject disturbances faster and with less deviation from the servo problem scenario. This is due to the fact that the variable pole adaptive formulation adjusts both the time scale parameters and gains during the set point transition, resulting in better disturbance rejection ability at the new operating point in the high-purity region.

**Table 4:** BD Column: Comparison of Closed-Loop Performance-Regulatory Problem

control scheme	*J*_**MPC**_	ISE (**x**_D_)	ISE (**x**_B_)
fixed pole AMPC	94.099	0.0024	0.008
variable pole AMPC	122.14	0.0002	0.0004

### 3.4 Case Study-02: Experimental Studies on Two-Tank Heater Setup

**Figure 13:**
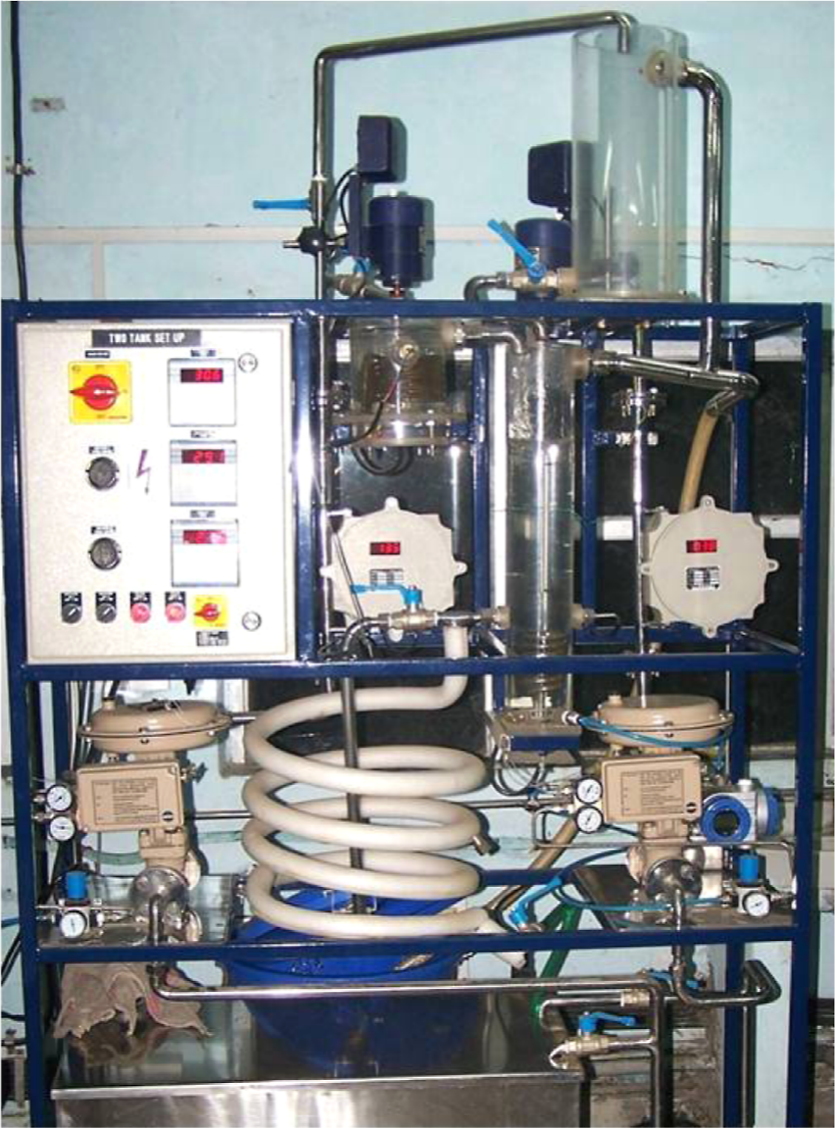
Pictorial view of the two-tank heater setup. Photograph courtesy of “Muddu Madakyaru”.

**Figure 14:**
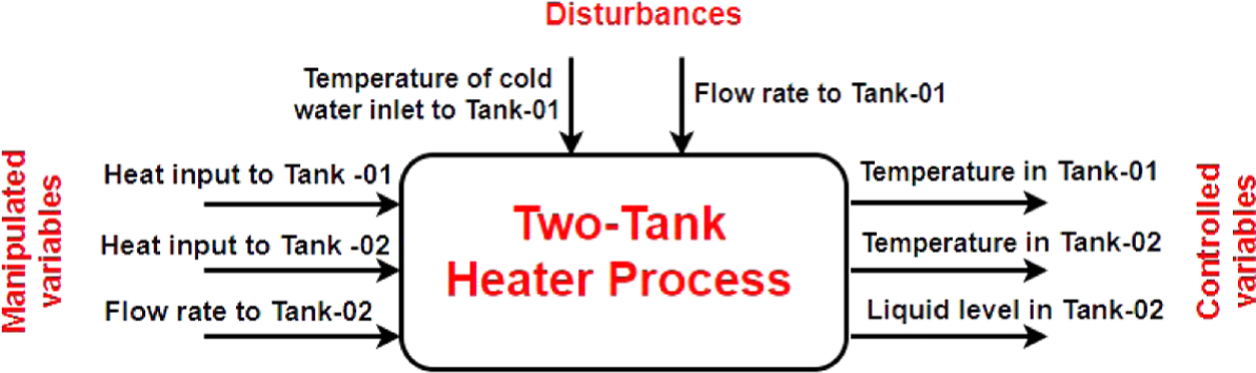
Block diagram indicating various variables of the two-tank heater setup.

This section deals with presenting model identification and AMPC closed-loop results using a benchmark two-tank heater setup^27^ located at the Automation Lab, Dept. of Chemical Engineering, IIT Bombay. A detailed description of the heater-mixer and schematic diagram can be found.^38^ In this study, first tank temperature (*T*_1_), second tank temperature (*T*_2_), and liquid level in the second tank (*H*_2_) are measured, and they act as controlled variables. The three manipulated inputs selected to regulate the controlled variable to keep it at the desired level as heat inputs to the first tank (**u**_1_), heat inputs to the second tank (**u**_2_), and flow of cold water to the second tank (**u**_3_). The pictorial view of the two-tank heater setup is shown in Figure 13, and a detailed description of various process variables is shown in Figure 14.

The nominal operating condition for the process can be found.^38^ The cold water inlet temperature (*T*_C_) acts like a drifting disturbance that slowly changes over the course of experimentation. The identification and validation data are generated by introducing PRBS signals to the input variables. To identify and validate a model, the process involves introducing simultaneous input perturbations (PRBS) into the system to generate data. Two separate datasets are obtained for model identification and validation purposes, respectively. During data collection, a sampling time of 5 s is used. To design the inputs for the setup, the “*idinput*” function in the system identification toolbox in MATLAB is utilized, and these signal details are provided in Table 5.

**Table 5:** Two-Tank Heater Setup: Details of Perturbation Signal

input	frequency band	magnitude of inputs
heat input 1 (*u*_1_)	[0 0.05]	[−846 W 610 W]
heat input 2 (*u*_2_)	[0 0.05]	[−623 W 448 W]
flow input (*u*_3_)	[0 0.03]	[−18 lph 28 lph]

The dataset consists of 1600 data points, out of which 900 data points were utilized for model identification (identification data), while the rest of the 700 data points were reserved to evaluate the model’s effectiveness (model validation).

### 3.5 Off-Line Model Identification

Two MISO and one SISO models were developed using an identification dataset. The models identified such that two poles between each input–output pair and the resulting optimal model parameters are listed in Table 6.

**Table 6:** Two-Tank Heater Setup: Optimum GOBF Model Parameters of the OBF–ARX Model

output	*u*_1_	*u*_2_	*u*_3_	**y**_*i*_
MISO—*T*_1_	[0.197 0.2002]	[0.0518 0.0449]	[0.0417 0.0319]	[0.8543 0.0530]
MISO—*T*_2_	[0.0674 0.0674]	[0.0242 0.0239]	[0.0398 0.0398]	[0.1218 0.0125]
SISO—*H*_2_			[0.4046 0.4046]	[15.6572]

**Figure 15:**
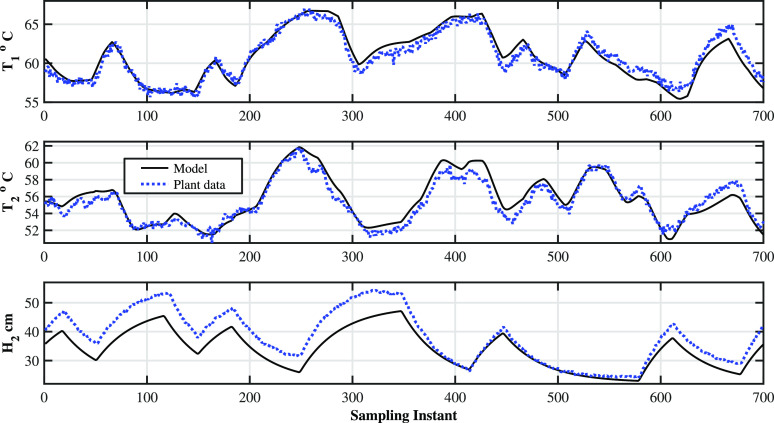
Two-tank heater setup: model validation—variations of model output and process data.

**Figure 16:**
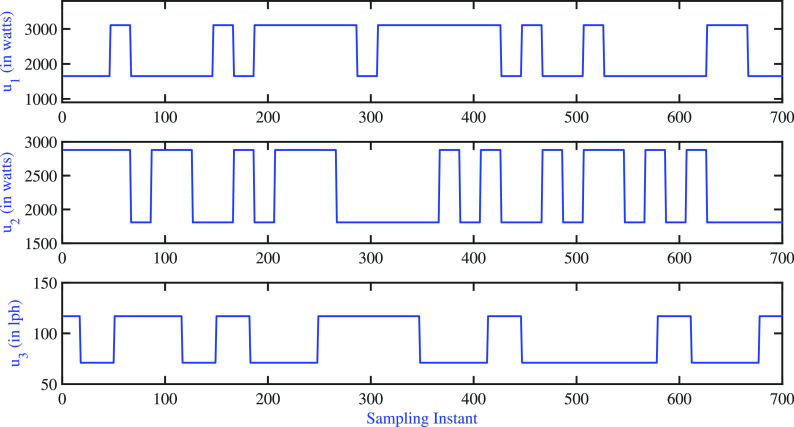
Two-tank heater setup: model validation—input variations.

**Figure 17:**
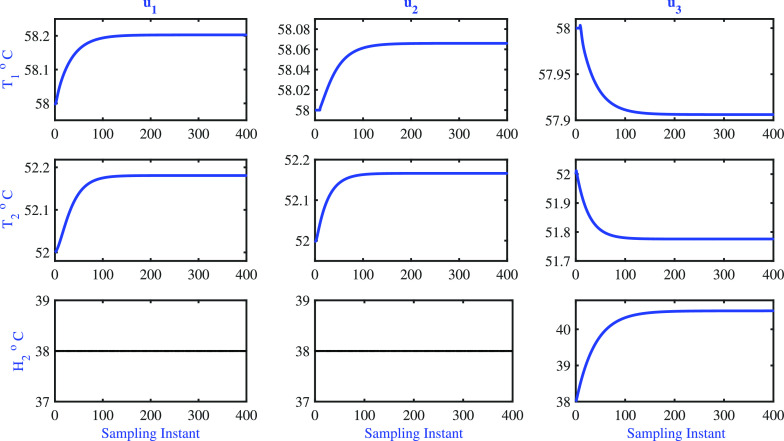
Two-tank heater setup: step response of the model.

The efficacy of the identified model is determined through dynamic validation using a testing dataset and the step response of the model. The dynamics validation results are shown in Figure 15, and the corresponding input moves are shown in Figure 16. The PPE values using validation and identification data are listed in Table 7. It is observed that high values of PPE values for output **y**_3_(=**H**_2_) are due to the fact that the initial bias between the model predictions and the process behavior. It is also clear that the model can predict the plant dynamics quite well (Figure 15), other than the bias. The step response shown in Figure 17 of the identified model gives better insight into the model. The step response clearly shows that the model is able to capture the right gain direction of the process.

**Table 7:** Two-Tank Heater Setup: PPE of Model Simulation

validation set	*T*_1_	*T*_2_	*H*_2_
model	9.0	13.9	36.21
identification set			
model	5.91	14.26	25.16

### 3.6 Closed-Loop Performance

This section demonstrates the closed-loop performance of the proposed AMPC scheme on the two-tank heater process. During the experimental study, 5 s was used as the sampling time. The AMPC formulation was transformed into quadratic optimization to evaluate the optimal control move. It is worth mentioning that the total computation was completed within a mere 1/10th of the sampling time. The set point tracking problem is attained by introducing simultaneous step changes in the temperature and liquid level variables and bringing the system back to the original set points. The input moves are constrained as follows
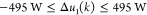
55
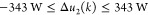
56
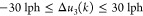
57It may be noted that the closed performance indices are reported after normalization using the number of data samples in the experimental run (***N***_s_), i.e., **ISE**(**y**_**i**_)/***N***_s_ and *J*_MPC_/***N***_s_ are reported. In addition, the settling time is evaluated for both AMPC formulations. The settling time is found using a ± 0.5 °C band around the final set point for temperature variables and ±0.5 cm band for the level.

#### 3.6.1 AMPC with Fixed Pole Location

**Figure 18:**
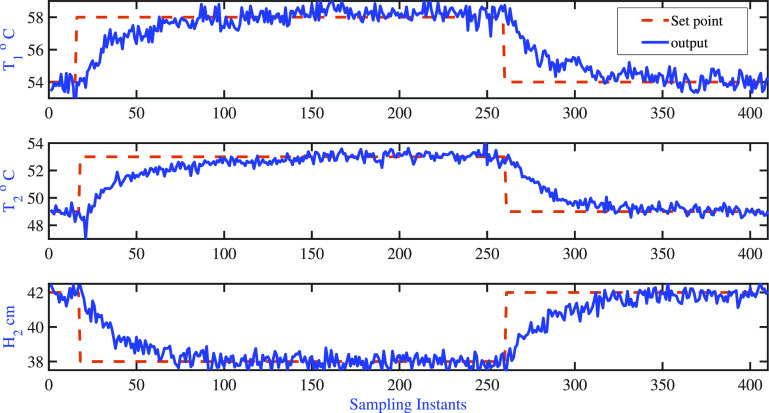
Two-tank heater setup: AMPC fixed poles: variations of the output variable.

**Figure 19:**
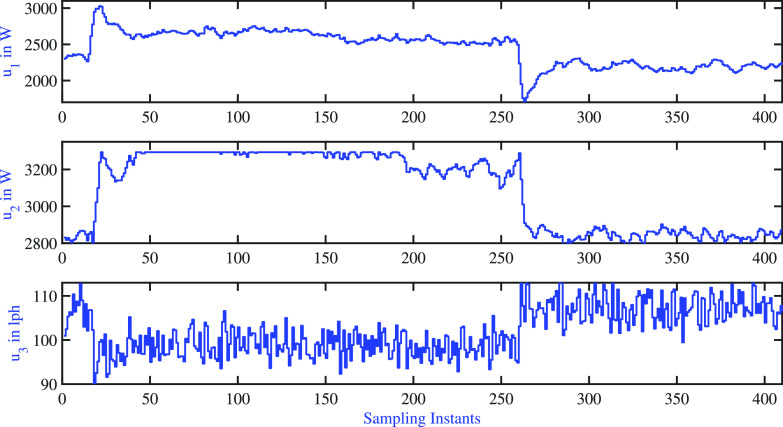
Heater-mixer setup: AMPC fixed poles: variations of the input variable.

The closed-loop response of fixed pole AMPC is shown in Figure 18, which represents the variations of controlled variables, and the corresponding manipulated input moves are shown in Figure 19. The tuning parameters are listed in Table 8. It is found that the open-loop settling time of the two-tank heater setup is approximately 60 samples with a sampling time of 5 s, which formed the basis for the choice of the prediction horizon in AMPC formulations. Since it is desired to achieve tight control of tank-01 and tank-02 temperatures, the error weighting matrix is selected as diag[1 1 1]. The control horizon is chosen as 5, and these degrees of freedom are distributed over the prediction horizon using four initial input blocks of 5 samples each and the final input block of 40 samples.

**Table 8:** Two-Tank Heater Setup: AMPC with Fixed Pole Location: Control Tuning Parameters

prediction horizon	60
control horizon	5
input blocking	[555540]
input weighting matrix	**I**_3×3_
error weighting matrix	diag[111]
filter coefficient	[0.95 0.95 0.9]

**Figure 20:**
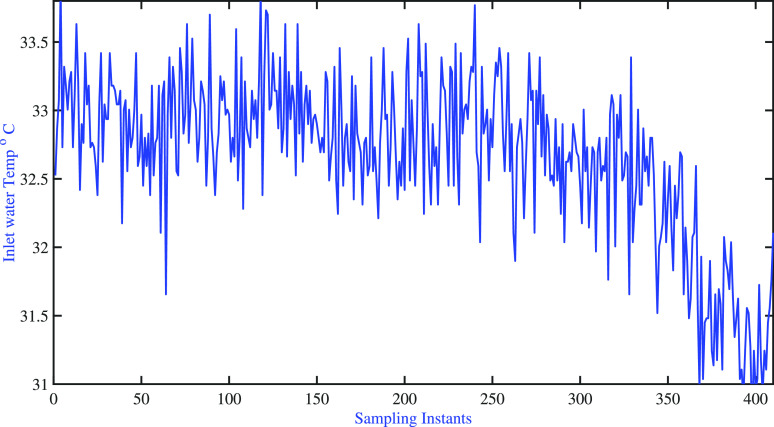
Two-tank heater setup: fixed pole AMPC: variations of unmeasured disturbance (i.e., inlet water temperature to tank-1 and tank-2).

**Figure 21:**
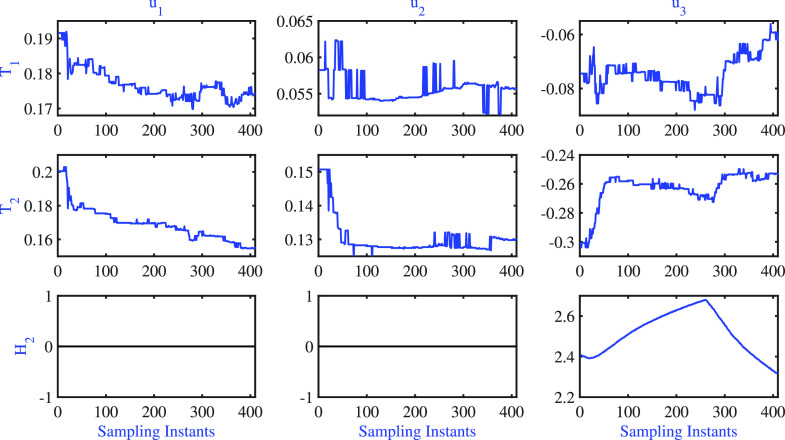
Two-tank heater setup: AMPC fixed pole: variations in model sensitivity.

It is observed that the cold water inlet temperature entering both tanks keeps changing around the nominal condition during the servo problem (see Figure 20). The adaptive model characteristics best explained through model sensitivity and variations are listed in Figure 21. The settling time details are listed in Table 9. Figure 21 clearly indicates the significant changes in the model as the operating conditions of the process varies. Also, it clearly shows that the proposed fixed pole AMPC is able to track the desired set point changes.

**Table 9:** Two-Tank Heater Setup: AMPC Fixed Pole: Settling Time for Servo Problem in Sampling Instant

	*T*_1_	*T*_2_	*H*_2_
step-1	75	135	85
step-2	100	72	80

#### 3.6.2 AMPC with Variable Pole

**Figure 22:**
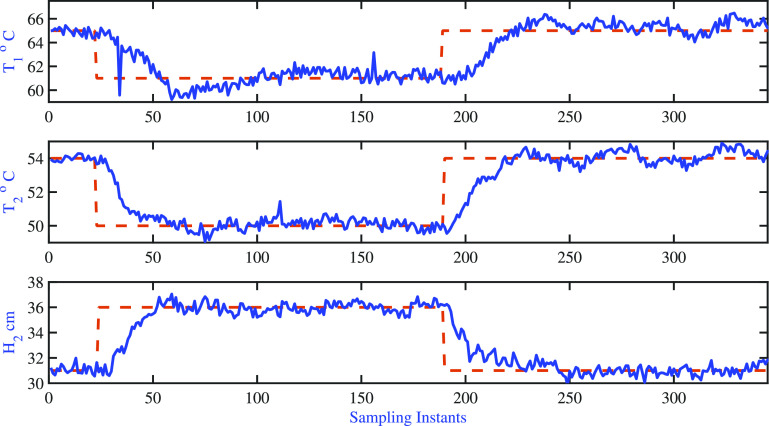
Two-tank heater setup: AMPC variable pole: variations of controlled variable.

**Figure 23:**
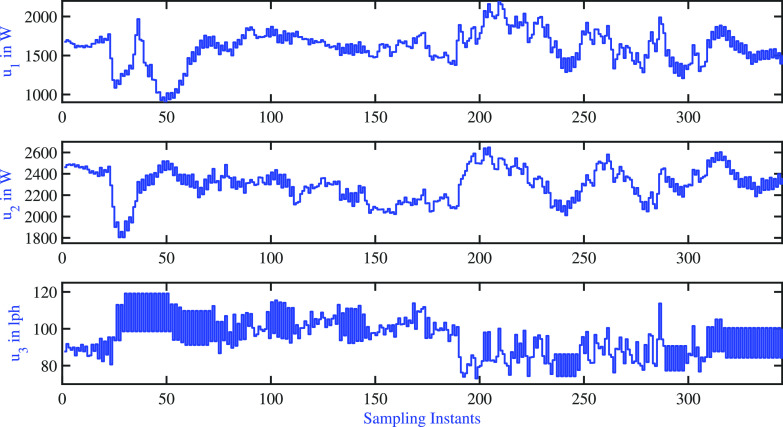
Two-tank heater setup: AMPC variable pole: variations of manipulated inputs.

In this subsection, the proposed closed-loop experiments demonstrated the two-tank process results of variable AMPC. The tuning parameters of the AMPC variable pole used for the closed-loop exercises are presented in Table 10. The variation of controlled variables is shown in Figure 22 for a sequence of step changes in all of the controlled variables. The corresponding variation of the manipulated variables is shown in Figure 23. As in the case of the AMPC fixed pole, the cold water inlet temperature to both tanks changes during the course of the regulatory problem, and these variations act as an unmeasured disturbance. The resulting settling time is listed in Table 11. It is observed that the AMPC variable pole closed-loop response settles faster than that of the AMPC fixed pole. However, similar to the simulation study, the variable pole AMPC resulted in larger input variability.

**Table 10:** Two-Tank Heater Setup: AMPC Variable Pole Tuning Parameters

prediction horizon	60
control horizon	1
input blocking	[60]
input weighting matrix	**I**_3×3_
error weighting matrix	**I**_3×3_
filter coefficient	[0.95 0.95 0.9]

**Table 11:** Two-Tank Heater Setup: AMPC Variable Pole: Settling Time for Servo Problem in Sampling Instant

AMPC type	*T*_1_	*T*_1_	*H*_2_
step-1	78	43	33
step-2	62	37	57

## 4 Conclusions

This study aims to synthesize AMPC that can adapt to changing conditions. The approach involves using state observers that are identified from input–output data, which are then parametrized by using GOBF. The parameters of the state-to-output map are updated online using a RLS approach. This leads to two different methods of online state estimation, which result in two parameter adaptation schemes: one where the poles are fixed but the gain matrix is variable, and another where both the poles and the gain matrix are variable.

To test the efficacy of these AMPC schemes, simulations were conducted on a BD column, and experimental evaluations were carried out on a benchmark two-tank heater setup. The results of the simulations indicated that the AMPCs provided satisfactory service and regulatory performance. However, it was found that the variable pole scheme resulted in higher variability of the manipulated inputs.

The experimental study confirmed the feasibility of these AMPC schemes, providing evidence of the effectiveness of both the fixed- and variable pole schemes. Overall, the results of this study suggest that the proposed AMPC formulations have the potential to be used in a variety of industrial control applications.

## References

[ref1] Qin S. J.; Badgwell T. A. A survey of industrial model predictive control technology. Control Eng. Pract. 2003, 11, 733–764. 10.1016/S0967-0661(02)00186-7.

[ref2] Morari M.; Lee J. Model predictive control: past, Present and Future. Comput. Chem. Eng. 1999, 23, 667–682. 10.1016/s0098-1354(98)00301-9.

[ref3] Ljung L. System Identification: Theory for the users; Prentice-Hall: New Jersy, 1999.

[ref4] Genceli H.; Nikolaou M. New Approach to Constrained Predictive Control with Simultaneous Model Identification. AIChE J. 1996, 42 (10), 2857–2868. 10.1002/aic.690421015.

[ref5] Clarke D. W.; Mohtadi C.; Tuffs P. Generalized Predictive Control: 1. The Basic Algorithm. Automatica 1987, 23 (2), 137–148. 10.1016/0005-1098(87)90087-2.

[ref6] Karra S.; Shaw R.; Patwardhan S. C.; Noronha S. Adaptive Model predictive Control of Multivariable Time-varying Systems. Ind. Eng. Chem. Res. 2008, 47, 2708–2720. 10.1021/ie070823y.

[ref7] Ydsti B. E. Certainty Equivalence Adaptive Control: What’s New in the Gap. In Chemical Process Control; Kantor J. C., Garcia C. E., Carnaham B., Eds.; CACHE-AIChE: Tahoe City, California, 1997; Chapter V, Vol. 46, pp 9–23.

[ref8] Seborg D. E.; Edgar T. F.; Shah S. L. Adaptive Control Strategies for Process Control: A Survey. AIChE J. 1986, 32 (6), 881–913. 10.1002/aic.690320602.

[ref9] Shouche M.; Genceli H.; Vuthandam P.; Nikolaou M. Simultaneous Constrained Model Predictive Control and Identification of DARX processes. Automatica 1998, 34 (12), 1521–1530. 10.1016/S0005-1098(98)80005-8.

[ref10] Zhu Y. Multivariable Process Identification for MPC: The Asymptotic Method and its Applications. J. Process Control 1998, 8 (2), 101–115. 10.1016/s0959-1524(97)00035-8.

[ref11] Nikolaou M.; Vuthandam P. FIR model identification: Parsimony through kernel compression with wavelets. AIChE J. 1998, 44 (1), 141–150. 10.1002/aic.690440115.

[ref12] Ohshima M.; Ohno H.; Hashimoto I.; Sasajima M.; Maejima M.; Tsuto K.; Ogawa T. Model predictive control with adaptive disturbance prediction and its application to fatty acid distillation column control. J. Process Control 1995, 5 (1), 41–48. 10.1016/0959-1524(95)95944-9.

[ref13] Mdoe Z.; Krishnamoorthy D.; Jäschke J. Stability properties of the adaptive horizon multi-stage MPC. J. Process Control 2023, 128, 103002–103015. 10.1016/j.jprocont.2023.103002.

[ref14] Griffith D. W.; Biegler L. T.; Patwardhan S. C. Robustly stable adaptive horizon nonlinear model predictive control. J. Process Control 2018, 70, 109–122. 10.1016/j.jprocont.2018.07.014.

[ref15] Boiroux D.; Bátora V.; Hagdrup M.; Wendt S. L.; Poulsen N. K.; Madsen H.; Jørgensen J. B. Adaptive model predictive control for a dual-hormone artificial pancreas. J. Process Control 2018, 68, 105–117. 10.1016/j.jprocont.2018.05.003.

[ref16] Maiti S. N.; Saraf D. N. Adaptive dynamic matrix control of a distillation column with closed loop online identification. J. Process Control 1995, 5 (5), 315–327. 10.1016/0959-1524(95)00002-8.

[ref17] Nouwens S. A. N.; Paulides M. M.; Heemels M. Constraint-adaptive MPC for linear systems: A system-theoretic framework for speeding up MPC through online constraint removal. Automatica 2023, 157, 111243 10.1016/j.automatica.2023.111243.

[ref18] Sasfi A.; Zeilinger M. N.; Köhler J. Robust adaptive MPC using control contraction metrics. Automatica 2023, 155, 111169 10.1016/j.automatica.2023.111169.

[ref19] Pereira G. C.; Wahlberg B.; Pettersson H.; Mårtensson J. Adaptive reference aware MPC for lateral control of autonomous vehicles. Control Eng. Pract. 2023, 132, 105403 10.1016/j.conengprac.2022.105403.

[ref20] Mdoe Z.; Krishnamoorthy D.; Jäschke J. Stability properties of the adaptive horizon multi-stage MPC. J. Process Control 2023, 128, 103002 10.1016/j.jprocont.2023.103002.

[ref21] Wang W.; Yan J.; Wang H.; Ge H.; Zhu Z.; Yang G. Adaptive MPC trajectory tracking for AUV based on Laguerre function. Ocean Eng. 2022, 261, 111870 10.1016/j.oceaneng.2022.111870.

[ref22] Sailo S.; Kumar K.; Patwardhan S. C.; Nataraj P.; Nataraj P. S. V. Development of Non-cooperative Distributed Adaptive MPC Schemes based on ARX Models parameterized using Orthonormal Basis Filters. IFAC-PapersOnLine 2021, 54, 257–262. 10.1016/j.ifacol.2021.08.251.

[ref23] Kumar K.; Patwardhan S. C.; Noronha S. An Adaptive dual MPC scheme based on using output Error Models Parameterized using Generalized Orthonormal Basis Filter. IFAC-PapersOnLine 2017, 50, 9077–9082. 10.1016/j.ifacol.2017.08.1644.

[ref24] Adetola V.; Guay M. Robust Adaptive MPC for Systems with Exogeneous Disturbances. IFAC Proc. Vol. 2009, 42, 249–254. 10.3182/20090712-4-TR-2008.00038.

[ref25] Liu Z.; Stursberg O. Recursive Feasibility and Stability of MPC with Time-Varying and Uncertain State Constraints. 18th European Control Conference; ECC: Napoli, Italy, 2019.

[ref26] Maiworm M.; Tobias B.; Rolf F. Scenario-based Model Predictive Control: Recursive Feasibility and Stability. IFAC-PapersOnLine 2015, 48, 50–56. 10.1016/j.ifacol.2015.08.156.

[ref27] Thornhill N. F.; Patwardhan S. C.; Shah S. L. A continuous stirred tank heater simulation model with applications. J. Process Control 2008, 18, 347–360. 10.1016/j.jprocont.2007.07.006.

[ref28] Sodderstrom T.; Stoica P. System Identification; Prentice-Hall: NJ, 1987.

[ref29] Ninness B. M.; Gustafsson F. A unifying construction of orthonormal basis for linear dynamical systems. IEEE Trans. Autom. Control 1996, 42 (4), 451–465. 10.1109/9.566661.

[ref30] Heuberger P. S. C.; Van Den Hof P. M. J.; Walhberg B. Modelling and Identification with Rational Orthogonal Basis Functions; Springer-Verlag: London, 2005.

[ref31] Patwardhan S. C.; Shah S. L. From data to diagnosis and control using generalized orthonormal basis filters. Part I: Development of state observers. J. Process Control 2005, 15 (7), 819–835. 10.1016/j.jprocont.2004.08.006.

[ref32] Srinivasarao M.; Patwardhan S. C.; Gudi R. D. From Data to Nonlinear Predictive Control: Part 1. Identification of Multivariable Nonlinear State Observers. Ind. Eng. Chem. Res. 2006, 45, 1989–2001. 10.1021/ie050904z.

[ref33] Ljung L.; Soderstrom T. Theory and Practice of Recursive Identification; MIT, Press: Cambridge, MA, 1983; p 11983.

[ref34] Muske K. R.; Rawlings J. B. Model Predictive Control with Linear Models. AIChE J. 1993, 39, 262–287. 10.1002/aic.690390208.

[ref35] Skogestad S.; postlethwaite I. Multivariable Feedback Control, 2nd ed.; Wiley: London, 2005.

[ref36] Muddu M.; Narang A.; Patwardhan S. C. Reparametrized ARX models for predictive control of staged and packed bed distillation columns. Control Eng. Pract. 2010, 18, 114–130. 10.1016/j.conengprac.2009.11.009.

[ref37] Pearson R. K.; Ogunnaike B. A. Nonlinear Process Identification. In Nonlinear Process Control; Henson M. A., Seborg D. E., Eds.; Prinitice hall PTR: Upper saddle river, NJ, 1997.

[ref38] Madakyaru M.; Patwardhan S. C. Adaptive predictive control using GOBF-ARX models: An experimental case study. IFAC Proc. Vol. 2013, 46 (32), 99–104. 10.3182/20131218-3-in-2045.00120.

